# Oxidation-Induced and Hydrothermal-Assisted Template-Free Synthesis of Mesoporous CeO_2_ for Adsorption of Acid Orange 7

**DOI:** 10.3390/ma15155209

**Published:** 2022-07-27

**Authors:** Yaohui Xu, Zhao Ding

**Affiliations:** 1 Laboratory for Functional Materials, School of New Energy Materials and Chemistry, Leshan Normal University, Leshan 614004, China; xyh1986@lsnu.edu.cn; 2 College of Materials Science and Engineering, National Engineering Research Center for Magnesium Alloys, Chongqing University, Chongqing 400044, China

**Keywords:** CeO_2_, mesoporous, template-free, hydrothermal, adsorption, azo dye

## Abstract

Hydrogen peroxide (H_2_O_2_), an accessible and eco-friendly oxidant, was employed for the template-free hydrothermal synthesis of mesoporous CeO_2_ based on a cerium carbonate precursor (Ce_2_(CO_3_)_3_•*x*H_2_O). Its microstructure and physicochemical properties were characterized by XRD, TEM and N_2_ sorption techniques. The formation of the CeO_2_ phase with a porous structure was strongly dependent on the presence of H_2_O_2_, while the values of the BET surface area, pore diameter and pore volume of CeO_2_ were generally related to the amount of H_2_O_2_ in the template-free hydrothermal synthesis. The BET surface area and pore volume of the mesoporous CeO_2_ synthesized hydrothermally at 180 °C with 10 mL H_2_O_2_ were 112.8 m^2^/g and 0.1436 cm^3^/g, respectively. The adsorption process had basically finished within 30 min, and the maximum adsorption efficiency within 30 min was 99.8% for the mesoporous CeO_2_ synthesized hydrothermally at 140 °C with 10 mL, when the initial AO7 concentration was 120 mg/L without pH preadjustment. The experimental data of AO7 adsorption were analyzed using the Langmuir and Freundlich isotherm modes. Moreover, the mesoporous CeO_2_ synthesized at 140 °C with 10 mL H_2_O_2_ was regenerated in successive adsorption–desorption cycles eight times without significant loss in adsorption capacity, suggesting that the as-synthesized mesoporous CeO_2_ in this work was suitable as an adsorbent for the efficient adsorption of AO7 dye from an aqueous solution.

## 1. Introduction

With the widespread use of various dyes, numerous dyes have been released into the environment in the process of the production and use of these dyes. Most dyes are extremely stable, and it is difficult for them to undergo natural degradation [1,2,3]. After entering a water environment, the chromaticity of the contaminated water is caused, which can affect the amount of incident light and the normal life activities of the aquatic animals and plants, and thus destruct the ecological balance of water. More severely, many dyes have carcinogenic and teratogenic effects because of their toxicity; they can directly or indirectly affect the health of the organism through the food chain [4,5,6,7]. Of today’s different groups of dyes, azo dyes are the most varied synthetic dyes, accounting for 80% of total organic dye products. The azo dye wastewater is recognized as an obstinate organic wastewater because of its stable chemical structure [8]. Therefore, how to get rid of azo dye pollution from wastewater has been attracting significant attention. So far, numerous technical and engineering approaches have been engaged to treat azo dye wastewater, such as the adsorption method using activated carbon [9], membrane separation technology [10], magnetic separation technology [11], the chemical oxidation method [12] and the biological method [13]. Among these techniques, adsorption using a suitable adsorbent is an alternative procedure and exhibits the best results [14]. Meanwhile, ceria (CeO_2_) with a mesoporous structure is a promising candidate for the removal of dye because of its high BET surface (*S*_BET_), well-defined pore topology and special surface states [15,16].

At present, most of the synthesis of CeO_2_ with porous structures involves the use of a series of soft or hard templates, and these templates must be sacrificed by subsequent dissolution using appropriate chemical reagents or decomposition by heat treatment [17,18,19]. For example, Jiang et al. [20] synthesized mesoporous CeO_2_ (*S*_BET_ = 150.8 m^2^/g) using the pre-synthesized SBA-15 molecular structure as a template. The binary composite material (CeO_2_/SBA-15) was first synthesized hydrothermally at 500 °C for 2 h. The as-synthesized CeO_2_/SBA-15 was added into 30 mL NaOH solution (10 mol/L) and stirred at 100 °C for 48 h. After that, the precipitates were rinsed with water to a pH of 7. The above steps were repeated two to three times, and finally the mesoporous CeO_2_ was obtained. Fu et al. [21] fabricated mesoporous CeO_2_ (*S*_BET_ = 107.9 m^2^/g) by the KIT-6-templating strategy using the ordered mesoporous silica (KIT-6) as a template. The mixture of KIT-6 and Ce(NO_3_)_3_•6H_2_O was calcined at 600 °C for 6 h, and the as-obtained powders were twice treated in a hot (60 °C) NaOH solution (2.0 mol/L) for the removal of the Si template. Recently, Li et al. [22] synthesized mono-dispersed hybrid microspheres composed of mesoporous CeO_2_ (*S*_BET_ = 67.2 m^2^/g) by the hydrothermal approach and controlled calcination procedure. Glucose and acrylamide were used as templates during the hydrothermal process, and the precursor obtained by hydrothermal process was first calcined at 600 °C for 6 h in an Ar atmosphere, and then calcined at 500 °C for 4 h in air. Moreover, Zhao et al. [23] synthesized mesoporous CeO_2_ nanospheres (*S*_BET_ = 77.8 m^2^/g) using the C-sphere template. The C-sphere@CeO_2_ precursor was first obtained by impregnation, combining the precipitation method with the prefabricated C-sphere as a template, and then 3D hollow mesoporous CeO_2_ nanospheres were obtained by calcining C-sphere@CeO_2_ precursor at 550 °C for 2 h. Although their template method could synthesize mesoporous CeO_2_, the soft or hard templates were essential, and thus either the procedure of dissolution or calcination was required to eliminate the soft or hard templates, but the energy consumption and costs were increased. Moreover, there have been limited reports for the direct and template-free synthesis of CeO_2_ with mesoporous structures until now [24,25,26]. Therefore, developing an effective, direct, and template-free synthetic strategy for mesoporous CeO_2_ is desirable. Despite their progresses in the template-free synthesis of mesoporous CeO_2_, it is still challenging to further simplify the process, reduce costs and reduce energy consumption.

Herein, mesoporous CeO_2_ was synthesized by the hydrothermal process without adding any precipitants and template agents, and the subsequent high temperature roasting process was eliminated. So, the process has the advantages of simple operation, low cost and low energy consumption. Compared with previous research work in 2015 [27], this study used the off-the-shelf commercial Ce_2_(CO_3_)_3_•*x*H_2_O instead of pre-synthesized Ce_2_(CO_3_)_3_•8H_2_O as the precursor, and studied the effects of the H_2_O_2_ addition amount and reaction temperature on the *S*_BET_ and adsorption efficiency of acid orange 7 (AO7, azo dye). The cerium carbonate precursor (Ce_2_(CO_3_)_3_•*x*H_2_O) was purchased and used as received without further purification. Significantly, hydrogen peroxide (H_2_O_2_), an accessible and eco-friendly oxidant, was employed to achieve the phase transformation of Ce_2_(CO_3_)_3_•*x*H_2_O to CeO_2_ with a mesoporous structure under the cooperation of the following hydrothermal treatment. The microstructure and physicochemical properties of samples were characterized by XRD, TEM and N_2_ adsorption–desorption analyses. Moreover, the adsorption abilities of the as-synthesized mesoporous CeO_2_ were evaluated by adsorptive removal of AO7.

## 2. Experimental

### 2.1. Starting Materials

Cerium carbonate (Ce_2_(CO_3_)_3_•*x*H_2_O, 99.9%) and acid orange 7 (AO7, >97.0%) were supplied by Shanghai Maclin Biochemical Technology Co., Ltd. (Shanghai, China) and Tokyo Chemical Industry Co., Ltd. (Shanghai, China), respectively. The general characteristics of the AO7 dye are shown in Table 1. Hydrogen peroxide (H_2_O_2_, ≥30%) and ethanol (≥99.7%) were supplied by Chengdu Kelong Chemical Co., Ltd. (Chengdu, China). All major chemicals were used as received without further purification, and distilled water was used in all experiments.

### 2.2. Synthesis

H_2_O_2_ was selected as an oxidant to assist the phase transformation of Ce_2_(CO_3_)_3_•*x*H_2_O precursor to CeO_2_, and the hydrothermal process was employed to synthesize the final product, CeO_2_ with a porous structure. Typically, 3 mmol Ce_2_(CO_3_)_3_•*x*H_2_O powders and the desired amount of H_2_O_2_ (2, 5, 8, 10 and 15 mL) were mixed, and the solution was allowed to stand for 2 h. After that, the distilled water was added to make a final volume of 20 mL. The above solution with precipitate was decanted into a 50 mL Teflon-lined stainless steel autoclave and maintained for 24 h at a set temperature (120, 140, 160, 180 and 200 °C). Finally, the pale yellow powders were collected and washed with distilled water and ethanol, and dried under air at 60 °C for 24 h.

For comparison, a sample was synthesized following the same procedure as the control at 180 °C for 24 h but in the absence of H_2_O_2_.

### 2.3. Characterization

The phases of the samples were examined by X-ray diffraction (XRD, DX-2700). The morphologies and microstructures of samples were examined by transmission electron microscopy (TEM, JEM-2100F). Nitrogen (N_2_) adsorption–desorption isotherms of CeO_2_ samples were measured on Micromeritics ASAP2460, and their specific surface areas (*S*_BET_) were calculated by the Brunauer–Emmett–Teller (BET) method. The pore diameters and pore volumes were determined by Barrett–Joyner–Halenda (BJH) analysis.

### 2.4. Evaluation of Adsorption Capacity

AO7 is a typical azo dye that is widely used in textile industries because of its low cost and high solubility in water. AO7 is a toxic synthetic dye, and its poor degradability allows it to exist in the environment for a long time and then cause environmental pollution. So, the removal of AO7 dye from water and wastewater due to its detrimental effects is essential. In this work, the adsorption ability of porous CeO_2_ was evaluated by the adsorptive removal of AO7 dye from simulated wastewater. Typically, 0.2 g of the as-synthesized CeO_2_ was dispersed into 100 mL AO7 solution with an initial concentration of 120 mg/L, and the mixture was stirred using a vibrator (200 rpm). About 4 mL of the suspension was taken continually at regular intervals and centrifuged. The absorbance of supernatant at regular intervals (*A_t_*, *a.u.*) was measured at the maximum absorption wavelength of 484 nm for AO7 dye using an ultraviolet spectrophotometer (Techcomp UV-2600), and the adsorption efficiency at this moment (*E_t_*, %) was estimated as the following Equation (1):

(1) $$E_{t}(\%)=\frac{A_{0}-At}{A_{0}}\times 100\;$$

where *A*_0_ is the initial absorbance value of AO7 dye solution ([AO7] = 120 mg/L) at the λ_max_ of 484 nm.

## 3. Results and Discussion

### 3.1. Characterization of Mesoporous CeO_2_

**Figure 1:**
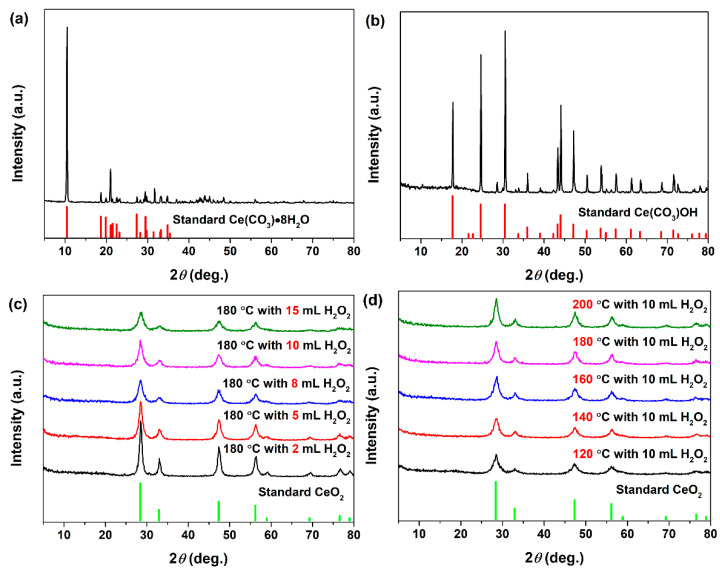
XRD patterns of (**a**) commercial Ce_2_(CO_3_)_3_•*x*H_2_O powders; The resulting precipitate synthesized hydrothermally (**b**) at 180 °C for 24 h without adding H_2_O_2_, (**c**) at 180 °C for 24 h with desired amounts H_2_O_2_ of 2–15 mL, and (**d**) at a set temperature of 120–200 °C for 24 h with 10 mL H_2_O_2_.

The phases of all samples were detected by XRD analysis. Figure 1a shows the XRD patterns of commercial Ce_2_(CO_3_)_3_•*x*H_2_O powders. As shown in Figure 1a, the XRD pattern of commercial Ce_2_(CO_3_)_3_•*x*H_2_O was well indexed to the characteristic peaks of Ce_2_(CO_3_)_3_•8H_2_O (Orthorhombic; JCPDS no. 38-0377), revealing the major chemical composition was Ce_2_(CO_3_)_3_•8H_2_O. Furthermore, the diffraction peaks at the diffraction angle in the 2*θ* region of 36–80° were not matched to any substance from JCPDS standard cards, but its profile was similar to these previous reports on Ce_2_(CO_3_)_3_•8H_2_O [27,28]. Figure 1b shows the XRD pattern of the resulting precipitate synthesized hydrothermally at 180 °C for 24 h without adding H_2_O_2_. The major phase of the as-obtained precipitate was Ce(CO_3_)OH (Hexagonal; JCPDS no. 52-0352). It could be found that pure CeO_2_ phase was not obtained hydrothermally in the absence of H_2_O_2_.

Figure 1c,d show the resulting precipitates synthesized hydrothermally at 180 °C with a desired amount of H_2_O_2_ and synthesized hydrothermally at a set temperature with 10 mL H_2_O_2_, respectively. As observed in Figure 1c,d, all broad peaks had a good match with the standard CeO_2_ pattern (Cubic; JCPDS no. 34-0394), suggesting that the as-synthesized CeO_2_ had a good crystallinity. Moreover, no additional phases for impurities were detected (such as Ce_2_(CO_3_)_3_•8H_2_O and Ce_2_(CO_3_)OH,), which indicated that the single phase CeO_2_ could be successfully obtained by hydrothermal process in the presence of H_2_O_2_. The FWHM (full width at half maximum) in Figure 1c showed obvious broadening phenomenon with the added volume of H_2_O_2_ increased. The broadening phenomenon of FWHM implied that the grain sizes of CeO_2_ decreased. In the formation process of the CeO_2_ phase, the H_2_O_2_ acts as an oxidant; their added volume directly affects the number of CeO_2_ crystal nucleus, and then affects their grain size. From Figure 1d, no significant changes on FWHM were observed with the increase in the hydrothermal temperature from 120 to 200 °C, which could be due to the constant amount of H_2_O_2_ (10 mL). The results showed that the addition amount of H_2_O_2_ could affect the grain size of the CeO_2_ final products. According to the above XRD results of the evolution process, a clear phase transformation from orthorhombic Ce_2_(CO_3_)_3_•8H_2_O to cubic CeO_2_ with better crystallinity was observed, which could verify the mechanism involving the oxidation-assisted dissolution of Ce_2_(CO_3_)_3_•*x*H_2_O precursor followed by the formation of the CeO_2_ phase.

**Figure 2:**
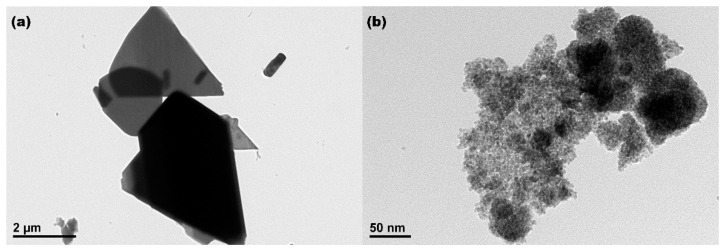
TEM images of (**a**) commercial Ce_2_(CO_3_)_3_•*x*H_2_O particles and (**b**) CeO_2_ sample synthesized hydrothermally at 200 °C for 24 h with 10 mL H_2_O_2_.

The morphologies, sizes and microstructures of commercial Ce_2_(CO_3_)_3_•*x*H_2_O precursor and CeO_2_ sample synthesized hydrothermally at 200 °C with 10 mL H_2_O_2_ were measured by TEM analysis. As observed in Figure 2a, there were no uniform morphologies and uniform sizes for commercial Ce_2_(CO_3_)_3_•*x*H_2_O particles, and these particles were basically on the micron scale with smooth and compact surfaces. After hydrothermal treatment at 200 °C in the presence of H_2_O_2_, it was clearly observed that the as-obtained CeO_2_ particles consisted of aggregated nanoparticles with a mean diameter of about 4.5 nm, and the pores resulted from these aggregated nanoparticles (see Figure 2b). This is a preliminary indication that the oxidation-induced and hydrothermal-assisted template-free synthesis of porous CeO_2_ is viable.

**Figure 3:**
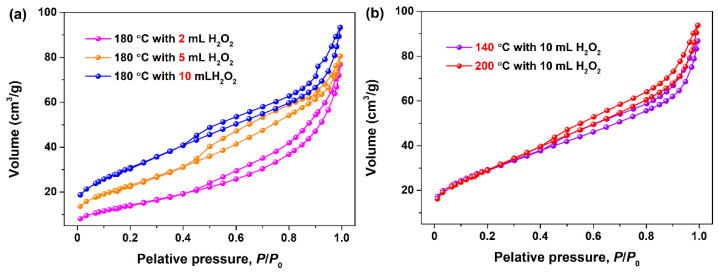
N_2_ adsorption–desorption isotherms of the mesoporous CeO_2_ synthesized hydrothermally (**a**) at 180 °C for 24 h with a desired amounts H_2_O_2_ of 2, 5 and 10 mL, and (**b**) the mesoporous CeO_2_ synthesized hydrothermally at a set temperature of 140 and 200 °C for 24 h with 10 mL H_2_O_2_.

To further clarify the porous nature of the CeO_2_ final products, N_2_ adsorption–desorption experiments were conducted, and their *S*_BET_, average pore sizes and pore volumes were estimated by N_2_ physisorption. Figure 3a,b show the N_2_ adsorption–desorption isotherms of the porous CeO_2_ synthesized hydrothermally at 180 °C with the desired amounts of H_2_O_2_ of 2, 5 and 10 mL, and at a set temperature of 140 and 200 °C with 10 mL H_2_O_2_, respectively. From Figure 3, the similar hysteresis loops in the relative pressure (*P*/*P*_0_) range of 0.4–1.0 were observed, and these N_2_ adsorption–desorption isotherms were consistent with that of the mesoporous CeO_2_ reported in literatures [29,30,31], suggesting that these as-obtained CeO_2_ belonged to the mesoporous material [32].

The determined values of *S*_BET_, pore diameters and pore volumes are summarized in Table 2. As observed in Table 2, the *S*_BET_ of the mesoporous CeO_2_ powders synthesized hydrothermally at 180 °C with 2, 5 and 10 mL H_2_O_2_ were determined as 52.5, 84.9 and 112.8 m^2^/g, respectively. These results implied that the amount of H_2_O_2_ played a decisive role on the *S*_BET_, as well as the pore diameter and pore volume. In other words, the more H_2_O_2_ added, the larger these physicochemical parameters. Meanwhile, it can be found that the *S*_BET_ of the mesoporous CeO_2_ synthesized hydrothermally at 140, 180 and 200 °C with 10 mL H_2_O_2_ were 107.0, 112.8 and 109.4 m^2^/g, respectively. It suggested that the hydrothermal temperature had little effect on the *S*_BET_ of the mesoporous CeO_2_ powders; however, it could affect the surface state of CeO_2_, such as the empty 4f orbital of the cerium ion onto the CeO_2_ surface. Combining with the results of the XRD and TEM analyses, we could derive a conclusion that H_2_O_2_ as an oxidant would play an important role in achieving phase transformation from Ce_2_(CO_3_)_3_•*x*H_2_O to CeO_2_ with a mesoporous structure; the addition amount of H_2_O_2_ not only affects the grain size of CeO_2_, but also determines the *S*_BET_, pore diameters and pore volumes.

### 3.2. Adsorption Characteristics

**Figure 4:**
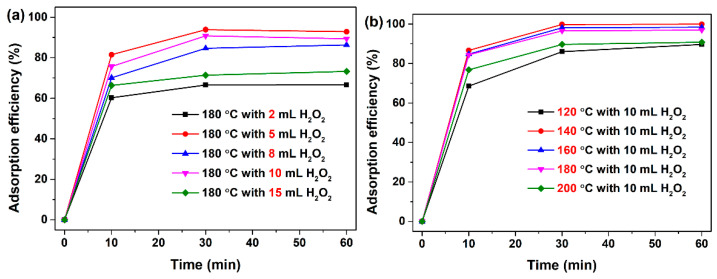
(**a**) Time-dependence of adsorption profiles of AO7 dye without pH pre-adjustment onto mesoporous CeO_2_ synthesized hydrothermally at 180 °C for 24 h with a desired amount H_2_O_2_ of 2–15 mL and (**b**) synthesized hydrothermally at a set temperature of 120–200 °C for 24 h with 10 mL H_2_O_2_. ([CeO_2_] = 2.0 g/L; [AO7] = 120 mg/L; *V* = 100 mL; distilled water; 200 rpm; room temperature).

An anionic dye, AO7, was selected as the modal target to evaluate the adsorption performance of the as-synthesized mesoporous CeO_2_ powders without pH preadjustment. As shown in Figure 4a,b, the adsorption efficiencies within the first 10 min were surprisingly fast for all mesoporous CeO_2_ samples, above 60% of the AO7 was adsorbed, particularly the mesoporous CeO_2_ synthesized hydrothermally at 140 °C for 24 h with 10 mL H_2_O_2_, and the adsorption efficiency could reach 86.7%. Moreover, the adsorption efficiencies showed almost no significant changes after 30 min, indicating that the adsorption process had basically finished within 30 min. The maximum adsorption efficiency within 30 min was obtained with 99.8% for the mesoporous CeO_2_ synthesized hydrothermally at 140 °C with 10 mL H_2_O_2_. The fast and excellent adsorption of the mesoporous CeO_2_ for AO7 dye could be explained by the following three aspects. First, the as-synthesized CeO_2_ with mesoporous structures possessed high *S*_BET_, which could provide for numerous sites for the adsorption of AO7, and then it increased their adsorption capacities. Second, the abundant pore structure of the mesoporous CeO_2_ was conducive to the transference of AO7 molecule toward the inside of this porous material, and then it increased the effectiveness of the contact between CeO_2_ adsorbent and AO7 adsorbate. Third, the strong adsorption toward AO7 may be attributed to the chelation interaction between the electron-rich groups (sulfonate group, SO_3_^−^) of the AO7 molecule and the empty 4*f* orbital of cerium ion onto CeO_2_.

Significantly, the mesoporous CeO_2_ synthesized hydrothermally at 140 °C (*S*_BET_ = 107.0 m^2^/g), 180 °C (*S*_BET_ = 112.8 m^2^/g) and 200 °C (*S*_BET_ = 109.4 m^2^/g) with 10 mL H_2_O_2_ possessed similar *S*_BET_ (see Table 2); however, their adsorption efficiencies for AO7 within 30 min exhibited varying degrees of difference, and the values were 99.8%, 90.8% and 89.7%, respectively. Moreover, the mesoporous CeO_2_ synthesized hydrothermally at 180 °C with 10 mL H_2_O_2_ exhibited a maximum *S*_BET_ of 112.8 m^2^/g from Table 2; however, its adsorption efficiencies for AO7 within 30 min was not the maximum among all as-synthesized mesoporous CeO_2_ powders. It indicates that the *S*_BET_ is not the only factor for the adsorption of AO7 dye onto mesoporous CeO_2_ in this study, if any, including the CeO_2_ surface state, such as the empty 4*f* orbital of cerium ion on the CeO_2_ surface.

CeO_2_ has selective adsorption for the anion dye with SO_3_^−^ groups, especially methyl orange (MO) and AO7 dyes [33,34]. In general, there are three coordination modes of SO_3_^–^ group: monodentate coordination, double dentate mononuclear coordination and bicentate biconuclear coordination. According to Deacon and Phillip’s theory and Bauer’s hypothesis, the wave-number distance between the peaks of asymmetric and symmetric vibration from the isolated SO_3_^–^ groups is larger than that of the adsorbed one, indicating that the SO_3_^–^ groups and Ce atoms form a tooth bridge integration [35]. According to the geometrical structure of AO7 molecule, when the adsorption reaction between AO7 and CeO_2_ occurs, the two oxygen atoms on SO_3_^–^ group will coordinate with the two Ce atoms on CeO_2_, and the nitrogen atom from the azo bond (-N=N-) also will interact with the Ce atoms in the appropriate position [36].

**Figure 5:**
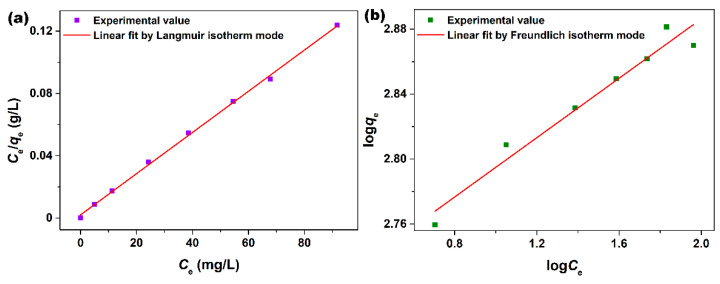
(**a**) Langmuir and (**b**) Freundlich linear fittings of AO7 molecule onto mesoporous CeO_2_ synthesized hydrothermally at 140 °C for 24 h with 10 mL H_2_O_2_.

To describe the interaction between the as-synthesized mesoporous CeO_2_ and AO7 molecule and investigate the adsorption mechanism, the experimental data were analyzed by the Langmuir (Equation (2)) [37] and Freundlich [38] (Equation (3)) isotherm models, as shown in Figure 5a,b.

(2) $$\frac{C_{e}}{q_{e}}=\frac{1}{q_{m}}C_{e}+\frac{1}{K_{L}q_{m}}$$

(3) $$\log q_{e}=\frac{1}{n}\log C_{e}+\log K_{F}$$

where *C*_e_ (mg/L) and *q*_e_ (mg/g) are the concentration of AO7 solution and the amount of AO7 adsorbed per gram of CeO_2_ at equilibrium, respectively. *q*_m_ (mg/g) is the maximum amount of AO7 molecule adsorbed per gram of CeO_2_. *K*_L_ and *K*_F_ are the Langmuir constant related to the energy of adsorption and the Freundlich constant related to the adsorption capacity, respectively. 1/*n* is the heterogeneity factor, and n is the adsorption intensity.

Figure 5a,b shows the Langmuir and Freundlich linear fittings of the experimental data of the adsorption of the AO7 molecule onto the mesoporous CeO_2_ synthesized hydrothermally at 140 °C with 10 mL H_2_O_2_, and the relevant parameters calculated by Langmuir and Freundlich linear fittings are listed in Table 3. As observed in Figure 5a,b, it is found that the adsorption of the AO7 molecule onto the mesoporous CeO_2_ can be described by both Langmuir and Freundlich isotherm models. However, the correlation coefficient (*R*^2^) for the Langmuir isotherm model (*R*^2^ = 0.9985) was much closer to 1.0 than that of the Freundlich isotherm model (*R*^2^ = 0.9512) from Table 3. According to the Langmuir isotherm model, the maximum amount of AO7 adsorbed on mesoporous CeO_2_ could reach 757.6 mg/g at room temperature. Moreover, the Freundlich adsorption constant (*n* = 10.94) related to the adsorption capacity was larger than 1, indicating that the adsorption intensity was favorable in the concentration range studied [39].

Table 4 shows the maximum amount (*q*_m_, mg/g) of AO7 molecule adsorbed per gram of various adsorbents from the recent literature [27,40,41,42,43,44,45,46,47,48,49,50,51,52]. By comparing the *q*_m_ of various adsorbent, we could see clearly that the adsorption capacity of the mesoporous CeO_2_ synthesized hydrothermally at 140 °C with 10 mL H_2_O_2_ in this work was among the very highest in these reported works in the literature. By noticing the *S*_BET_ and *q*_m_ of these adsorbents, it further indicated that the *S*_BET_ of the adsorbents was not the main factor determining their adsorption capacities. So, considering the unique electronic structure of CeO_2_, the adsorption mode of AO7 molecule on CeO_2_ surface could be described as a Lewis acid-based reaction between the SO_3_^−^ groups of AO7 molecule and empty 4*f* orbital of cerium ion on CeO_2_ surface, which eventually formed an inner-sphere complex. Therefore, both the addition amount of H_2_O_2_ and the hydrothermal temperature affected the physicochemical state of the CeO_2_ surface, and their joint action ultimately determined the adsorption capacity of mesoporous CeO_2_ for AO7 dye.

### 3.3. Desorption and Reusability

**Figure 6:**
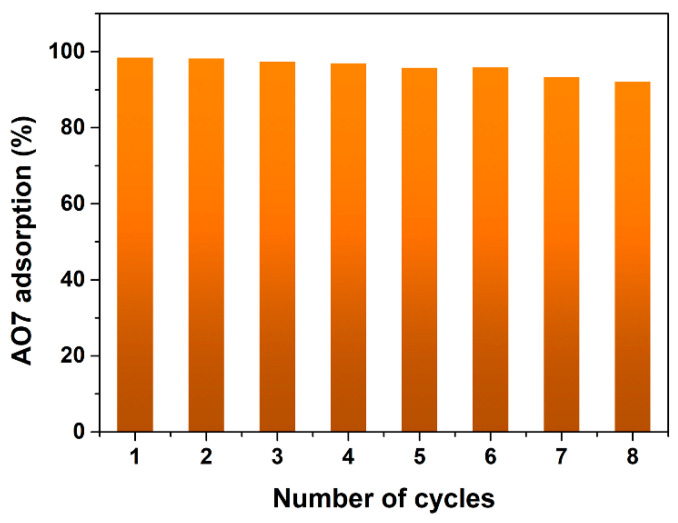
Adsorption histogram in successive adsorption–desorption cycles eight times. (Sample: mesoporous CeO_2_ powders synthesized hydrothermally at 140 °C for 24 h with 10 mL H_2_O_2_; desorbing agents: 20 mL 0.5 mol/L NaOH; desorption time: 5 min; room temperature).

Desorption of AO7 molecules from the adsorbed mesoporous CeO_2_, and the reusability of mesoporous CeO_2_ are essential. In this experiment, 0.5 mol/L NaOH solution was used to desorb AO7 molecules from the mesoporous CeO_2_ surface. The adsorption histogram in eight successive adsorption–desorption cycles is shown in Figure 6. It was clear that the adsorption efficiency could reach 98.4% in the first adsorption–desorption cycle. To examine the reproducibility of mesoporous CeO_2_, another seven adsorption–desorption cycles were performed. It can be found that the similar AO7 uptake capacity of the regenerated mesoporous CeO_2_ only appeared to be slightly fading, and the adsorption efficiency for AO7 could maintain more than 92% after eight cycles. Due to the high recycling efficiency, the as-synthesized mesoporous CeO_2_ in this work may be suitable as a promising absorbent for water treatment or the removing of the AO7 dye.

## 4. Conclusions

In summary, an oxidation-induced strategy was developed for the template-free hydrothermal synthesis of CeO_2_ with a mesoporous structure, in which commercial Ce_2_(CO_3_)_3_•*x*H_2_O was purchased and served as a cerium precursor, while H_2_O_2_ served as an accessible and eco-friendly oxidant employed to achieve the phase transformation of the Ce_2_(CO_3_)_3_•*x*H_2_O precursor to the CeO_2_ phase with a mesoporous structure under the cooperation of following the hydrothermal treatment. H_2_O_2_ as an oxidant had a decisive influence on the formation of cubic CeO_2_ phase as well as its mesoporous structure; moreover, the values of *S*_BET_, pore diameters and pore volumes were generally related to the amount of H_2_O_2_ in the template-free hydrothermal synthesis. The oxidation-induced and hydrothermal-assisted template-free synthesis of mesoporous CeO_2_ can be expected to provide a synthetic alternative for other porous inorganic materials. Preliminary adsorbate evaluation suggested that the as-synthesized mesoporous CeO_2_ was a promising absorbent for wastewater treatment containing AO7 dye; the maximum AO7 adsorption efficiency of these mesoporous CeO_2_ was found to be 99.8% within 30 min when the initial AO7 concentration was 120 mg/L without the pH preadjustment. The Langmuir isotherm fitted (*R*^2^ = 0.9985) the equilibrium data better than the Freundlich isotherm (*R*^2^ = 0.9512), with a higher correlation coefficient (*R*^2^). The maximum uptake capacity for mesoporous CeO_2_ was 757.6 mg/g for AO7 at room temperature according to the Langmuir isotherm model, and it could be easily regenerated by an alkali washing. Moreover, the regeneration experiments revealed the good potential of mesoporous CeO_2_ for reuse, even though a slight decrease in adsorption capacity was observed in the subsequent eight cycles.

## Figures and Tables

**Table 1:** General characteristics of AO7 dye.

Generic Name	Chemical Formula	Chemical Structure	Molecular Weight (g/mol)	Cas Number	λ_max_ (nm)	Appearance
Acid orange 7	C_16_H_11_N_2_NaO_4_S	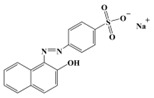	350.3	633-96-5	484	Orange-red

**Table 2:** Physicochemical properties of the mesoporous CeO_2_ synthesized hydrothermally at 180 °C for 24 h with a desired amounts H_2_O_2_ of 2, 5 and 10 mL, and the mesoporous CeO_2_ synthesized hydrothermally at a set temperature of 140 and 200 °C for 24 h with 10 mL H_2_O_2_.

Synthesis Conditions	180 °C with Desired Amounts of H_2_O_2_			Different Temperaments with 10 mL H_2_O_2_	
	2 mL	5 mL	10 mL	140 °C	200 °C
***S*_BET_ (m^2^/g)**	52.5	84.9	112.8	107.0	109.4
**Pore diameter (nm)**	8.95	5.81	5.09	4.98	5.28
**Pore volume (cm^3^/g)**	0.1174	0.1234	0.1436	0.1332	0.1445

The specific surface areas were calculated by Brunauer–Emmett–Teller (BET) method (labeled as *S*_BET_), while the pore diameters and pore volumes were determined by Barrett–Joyner–Halenda (BJH) analysis.

**Table 3:** Estimated parameters of Langmuir and Freundlich linear fittings for the adsorption of AO7 molecule onto mesoporous CeO_2_ synthesized hydrothermally at 140 °C for 24 h with 10 mL H_2_O_2_ at room temperature.

Langmuir Isotherm Model			Freundlich Isotherm Model		
*q*_m_ (mg/g)	*K*_L_	*R* ^2^	*n*	*K*_F_	*R* ^2^
757.6	0.6256	0.9985	10.94	505.3	0.9512

**Table 4:** Recent literature on adsorbent development for the adsorption of AO7 dye.

Authors	Adsorbent Name	Sorption Conditions	*S*_BET_ (m^2^/g)	*q*_m_ (mg/g)
Pedro Silva [40]	Spent brewery grains (SBG)	30 °C	/	30.5
Hamzeh [41]	Canola stalks (CS)	25 °C; pH = 2.5	/	25.1
Ashori [42]	Soybean stalk (SS)	25 °C; pH = 2.0	/	17.5
Lin [43]	Iron oxide-loaded biochar (Fe-BC) from sorghum straw	25 °C; pH = 6.0; 180 rpm	216.6	59.3
Noorimotlagh [44]	Mesoporous activated carbon prepared from Iranian milk vetch	pH = 7.0	565	99.0
Lim [45]	Zeolite-activated carbon macrocomposite	Room temperature; pH = 7.0	84.7	0.19
Aber [46]	Powdered activated carbon	25 °C; pH = 2.8	/	440
Jia [47]	Multi-walled carbon nanotubes (MWCNTs)	pH = 7.0	~1800	47.7 ± 0.79
Nourmoradi [48]	Activated carbon coated with zinc oxide (AC-ZnO)	25 °C	/	66.2
Ghasemi [49]	Zeolitic imidazolate framework-8 (ZIF-8)	25 °C; pH = 6.0; 200 rpm	978	80.5
Zhou [50]	Fe_3_O_4_-poly(methacryloxyethyltrimethyl ammonium chloride) (Fe_3_O_4_-pDMC)	pH = 3.0; 150 rpm	35.7	270.3
Huo [51]	Nickel (II) oxide (NiO)	25 °C; pH = 5.5	251.8	178.6
Li [52]	Amine shield-introduced-released porous chitosan hydrogel beads (APCB)	30 °C; 150 rpm	/	2571.0 (pH = 2.0); 363.6 (pH = 4.0)
Xu [27]	Mesoporous CeO_2_ synthesized based on integrating bottom-up and top-down routes in the previous report	25 °C; No pH preadjustment; 200 rpm	166.5	510.2
Xu	Mesoporous CeO_2_ synthesized hydrothermally at 140 °C for 24 h with 10 mL H_2_O_2_ in this work	Room temperature; No pH preadjustment; 200 rpm	107.0	757.6

## Data Availability

Not applicable.
